# Immune Exclusion Is Frequent in Small-Cell Carcinoma of the Bladder

**DOI:** 10.1155/2019/2532518

**Published:** 2019-05-02

**Authors:** Tim Mandelkow, Niclas C. Blessin, Eva Lueerss, Laura Pott, Ronald Simon, Wenchao Li, Björn Wellge, Nicolaus F. Debatin, Doris Höflmayer, Jakob R. Izbicki, Franziska Büscheck, Andreas M. Luebke, Corinna Wittmer, Frank Jacobsen, Florian Lutz, Eike Burandt, Stefan Steurer, Guido Sauter, Maria Christina Tsourlakis, Waldemar Wilczak, Andrea Hinsch, Sarah Minner

**Affiliations:** ^1^Department of Pathology, University Medical Center Hamburg-Eppendorf, Germany; ^2^Department of General, Visceral and Thoracic Surgery, University Medical Center Hamburg-Eppendorf, Germany

## Abstract

Small-cell cancer of the urinary bladder is a rare but highly aggressive disease. It is currently unclear whether immune checkpoint therapies that have been approved for urothelial carcinomas will also be efficient in small-cell carcinomas. In this study, we analyzed potential predictors of response including PD-L1 expression and the quantity and location of tumor-infiltrating lymphocytes (TILs) in 12 small-cell and 69 “classical” urothelial cancers by immunohistochemistry. The analysis revealed that small-cell carcinomas were characterized by the virtual absence of PD-L1 expression and an “immune-excluded” phenotype with only a few TILs in the center of the tumor (CT). In small-cell carcinomas, the average immune cell density in the CT (CD3: 159 ± 206, CD8: 87 ± 169 cells/mm^2^) was more than 3 times lower than that in the urothelial carcinomas (CD3: 625 ± 800, *p* < 0.001; CD8: 362 ± 626 cells/mm^2^, *p* = 0.004) while there was no significant difference in the immune cell density at the invasive margin (IM) (small-cell carcinomas CD3: 899 ± 733, CD8: 404 ± 433 cells/mm^2^; urothelial carcinomas CD3: 1167 ± 1206, *p* = 0.31; CD8: 582 ± 864 cells/mm^2^, *p* = 0.27). Positive PD-L1 staining was found in 39% of urothelial cancers, but in only 8% of small-cell bladder cancer cases (*p* = 0.04). Concordant with these data, a sharp decrease of PD-L1 positivity from >80% to 0% positive cells and of TILS in the CT from 466-1063 CD3-positive cells/mm^2^ to 50-109 CD3-positive cells/mm^2^ was observed in two cancers with clear-cut progression from “classical” urothelial to small-cell carcinoma. In conclusion, these data demonstrate that small-cell bladder cancer commonly exhibits an immune-excluded phenotype.

## 1. Introduction

Small-cell cancer of the urinary bladder represents a rare bladder cancer subtype accounting for about one percent of urinary bladder cancers [1, 2]. They are characterized by early metastasis and a particularly poor prognosis [2]. Treatment usually includes one of chemotherapy, radiotherapy, or cystectomy, although there is no accepted standard treatment for this cancer type [2]. Despite therapy, about 80% of patients die within 5 years after diagnosis [1, 3].

Immune checkpoint inhibitors represent a new and promising therapeutic option for a variety of cancer types, including urinary bladder cancer. Several immune checkpoint inhibitors have recently been approved by the FDA for therapy of refractory or metastatic urothelial tumors [4]. The successful treatment of a patient with metastatic small-cell bladder carcinoma with pembrolizumab [5], a programmed cell death protein 1 (PD-1) inhibitor, has led to an ongoing phase II study (NCT03430895) to evaluate anti-checkpoint therapies in patients with rare bladder cancer entities including small-cell carcinoma.

Expression of programmed cell death-ligand 1 (PD-L1) in tumor cells and cancer-associated inflammatory cells is one of the best established predictive parameters for response to current anticheckpoint therapies in cancer [6, 7]. A growing number of reports show that the quantity and location of leucocytes, particularly CD8^+^ T lymphocytes, relative to the cancer cells may also be relevant to understand the interaction of the immune system with a cancer [8–10]. The most commonly used classifications to describe patterns of immune cell infiltration include the “inflamed” (tumor rich in tumor-infiltrating lymphocytes), the “immune-excluded” (presence of immune cells at the invasive margin but absence of immune cells in the center of the tumor), and the “immune-desert” phenotype (absence of relevant numbers of immune cells both at the periphery and in the center of the tumor) [11, 12].

To understand the spatial composition and distribution of immune cells in small-cell bladder carcinoma, we investigated the PD-L1 status and the T cell infiltration patterns in 12 small-cell bladder carcinomas and compared these findings with data from 69 “classical” urothelial carcinomas.

## 2. Material and Methods

### 2.1. Patients and Tissues

Formalin-fixed paraffin-embedded tumor tissue samples from 12 patients with small-cell bladder carcinomas and 69 patients with urothelial carcinomas of the bladder were retrieved from the archives of the Institute of Pathology of the University Medical Center Hamburg-Eppendorf. The usage of archived diagnostic leftover tissues and clinical data from anonymized patients and their analysis for research purposes has been approved by local laws (HmbKHG, §12a).

### 2.2. Immunohistochemistry

Freshly cut consecutive conventional large tissue sections were stained for CD3, CD8, PD-1, and PD-L1 at the same day. Slides were deparaffinized and exposed to heat-induced antigen retrieval for 5 minutes in an autoclave at 121°C in pH 6 antigen retrieval buffer (Leica Biosystems, Wetzlar, Germany; #AR9961) for PD-1 and pH 9 retrieval buffer (Leica Biosystems, Wetzlar, Germany; # AR9640) for CD3, CD8, and PD-L1. Primary antibody specific for CD3 (rabbit polyclonal antibody, Dako, Santa Clara, US; #IR503; undiluted), CD8 (mouse monoclonal antibody, Dako, Santa Clara, US; #IR623; undiluted), PD-1 (mouse monoclonal [NAT105] antibody, Abcam, Cambridge, UK; #ab52587; 1 : 50), and PD-L1 (rabbit monoclonal [E1L3N®] antibody, Cell Signaling, Danvers, Massachusetts; #13684; 1 : 200) was applied at 37°C for 60 minutes. Bound antibody was then visualized using the EnVision Kit (Dako, Glostrup, Denmark) according to the manufacturer's directions.

### 2.3. Definition of Compartments and Quantification of CD3, CD8, and PD-1 Immunostaining

Digital images of stained slides were acquired using Leica's Aperio VERSA 8 automated microscope. The invasive margin (IM) and the center of the tumor (CT) were defined in each digital image. IM was defined as the area expanding 300 *μ*m into the stroma and 50 *μ*m into the tumor measured from the stromal tumor borderline. CT was defined as an area remote from the stromal tumor borderline in the depth of the tumor bulk. All areas matching these criteria were included in the analysis of each slide, except from areas with obvious staining artefacts or damaged tissue that were excluded from further analysis. Image analysis of the IM and CT areas was performed using ImageScope software package (Leica Microsystems, Wetzlar, Germany). The number of stained cells and the size of the measured region were recorded in each area of IM and CT, and the density of stained cells (number of cells per square mm) was calculated from these data.

### 2.4. Analysis of PD-L1 Immunostaining

For each cancer, the percentage of PD-L1-positive cancer and inflammatory cells was recorded. Tumors showing detectable PD-L1 staining in ≥1% of tumor cells were considered positive [13].

### 2.5. Statistics

JMP Pro 12 software package (SAS Institute Inc., NC, USA) and R version 3.4.3 (The R Foundation) [14] were used to plot the data, to calculate chi-square *p* values, to perform analysis of variance (ANOVA), and to calculate Pearson's correlation coefficient (*ρ*) in order to explore differences of cell densities between the IM and the CT.

## 3. Results

### 3.1. T Cell Density in Small-Cell Bladder and Urothelial Carcinomas

**Figure 1:**
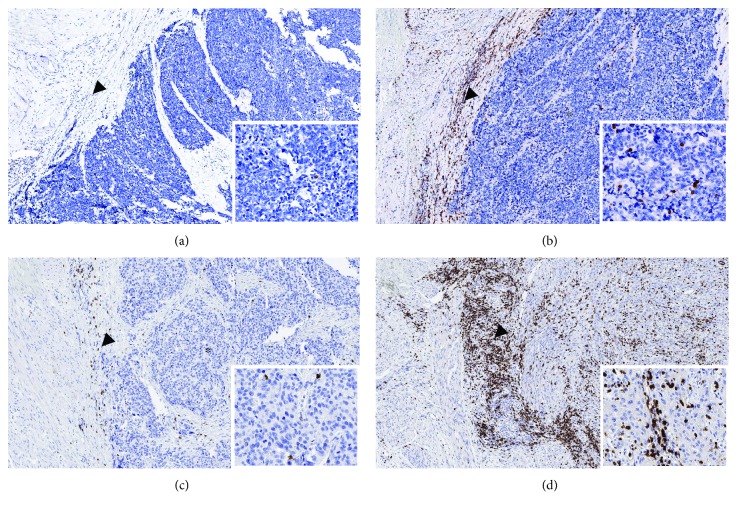
Representative pictures of CD3 staining (brown color) in (a, b) two cases of small-cell and (c, d) two cases of urothelial carcinoma of the bladder. Panels (a, c) show examples of the immune-desert phenotype and (b, d) examples of the immune-excluded phenotype. Arrowheads indicate the invasive margin. Asterisks label the center of the tumor, shown also in the insets at 400x magnification.

All 12 small-cell carcinomas and 69 urothelial carcinomas contained lymphocytes positive for CD3, CD8, and PD-1 at the invasive margin (IM) and in the center of the tumor (CT). Representative images with different densities of CD3^+^ T cells at the IM and in the CT are shown in Figure 1.

**Figure 2:**
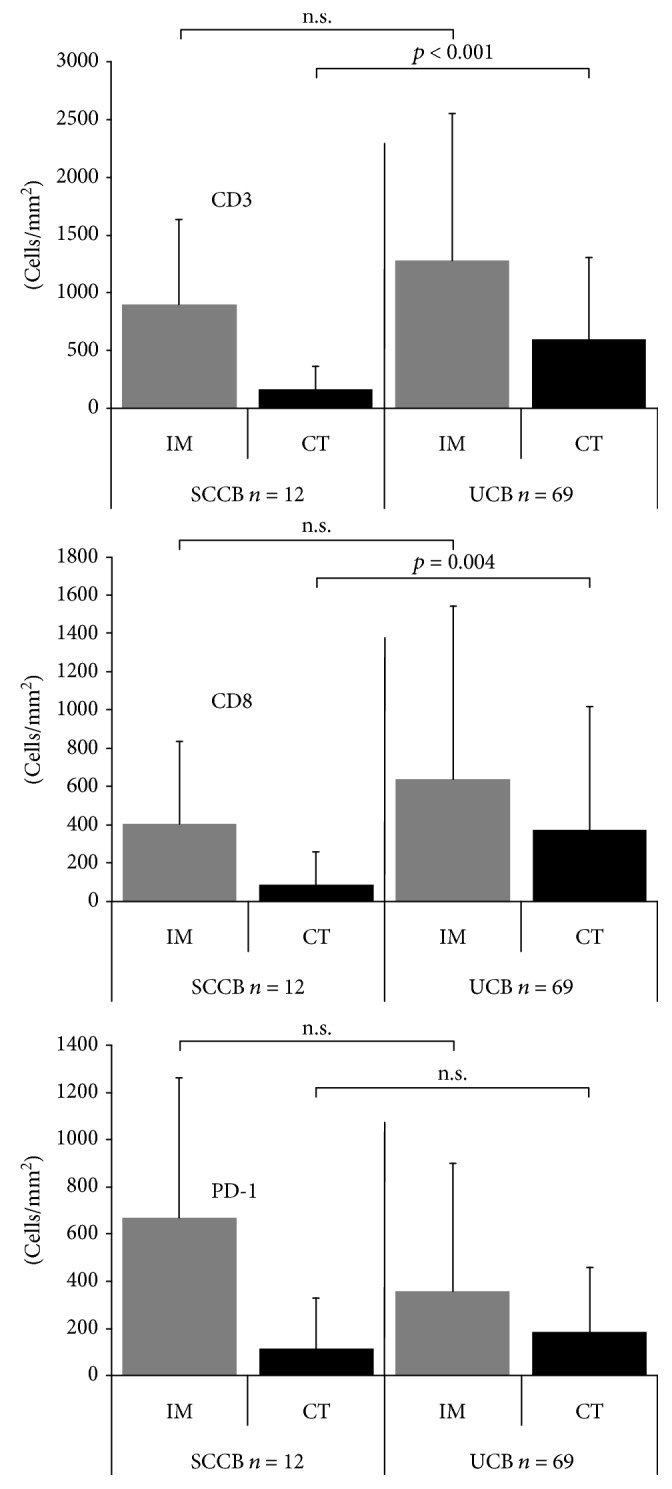
Comparison of CD3-, CD8-, and PD-1-positive cell densities at the invasive margin (IM) and center of the tumor (CT) between 12 small-cell carcinomas (SCCB) and 69 urothelial carcinomas of the bladder (UCB).

In small-cell carcinomas, the density of T cells was variable between patients but always markedly lower in CT than at IM (*p* < 0.05 each). This held true for CD3-positive cells/mm^2^ (IM: 899 ± 733, CT: 159 ± 206), CD8-positive cells/mm^2^ (IM: 404 ± 433, CT: 87 ± 169), and for PD-1-positive cells/mm^2^ (IM: 667 ± 595, CT: 215 ± 115). These differences were less evident in urothelial carcinomas. This was true for all T cell types including CD3-positive cells/mm^2^ (IM: 1167 ± 1206, CT: 625 ± 800), CD8-positive cells/mm^2^ (IM: 582 ± 864, CT: 362 ± 626), and PD-1-positive cells/mm^2^ (IM: 334 ± 517, CT: 208 ± 349). Further comparisons revealed that the T cell density in the CT of small-cell cancers was significantly lower than that of urothelial cancers (CD3: *p* < 0.001, CD8: *p* = 0.004), while there were no significant differences at the IM (CD3: *p* = 0.31, CD8: *p* = 0.27). All data are shown in Figure 2 and Supplementary Figure 1.

### 3.2. PD-L1 Status in Small-Cell and Urothelial Bladder Cancer

Two cases were excluded from this analysis because PD-L1 staining was not unequivocally interpretable. PD-L1 staining was less frequent and less intense in small-cell cancer than that in urothelial bladder cancer. Positive PD-L1 staining was found in the tumor cells of 26/67 (39%) urothelial carcinomas but in only 1/12 (8%, *p* = 0.04) of small-cell carcinomas. The one PD-L1-positive small-cell bladder cancer had 10% PD-L1-positive tumor cells. This was markedly less than the average fraction of PD-L1-positive tumor cells seen in 26 PD-L1-positive urothelial carcinomas (42% ± 36%).

### 3.3. Mixed Tumors with Small-Cell and Urothelial Cancer Components

**Figure 3:**
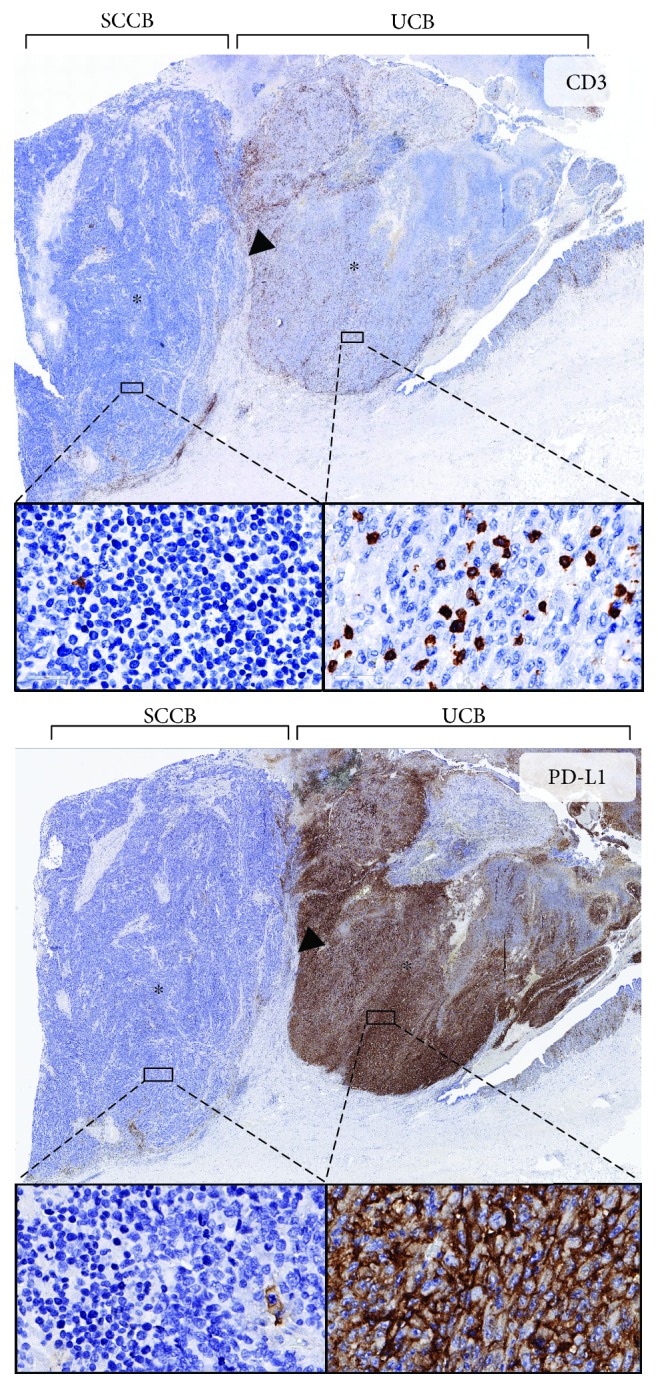
CD3 and PD-L1 staining of a sample from patient 4 that contained adjacent areas of small-cell and urothelial carcinoma of the bladder. Note the invasive margin (arrowhead) and the center of the tumor (asterisks). Insets show 400x magnification of the tumor bulk.

Our cohort included two cases that contained adjacent areas of small-cell and urothelial carcinoma (Figure 3). Both cases showed a dramatically lower T cell density in the CT of SCCB than that in the CT of UCB irrespective of the analyzed markers (CD3, CD8, and PD-1; Table 1). Again, no differences were found for the T cell densities in the IMs of both bladder cancer proportions. In addition, both cases showed strong PD-L1 staining in 80% and 90% of cells in the urothelial cancer component while PD-L1 staining was fully absent in the small-cell cancer fraction (Figure 3).

## 4. Discussion

The data from this study identify “immune exclusion” as the dominant immune pattern of small-cell carcinoma of the urinary bladder.

The typical features of immune exclusion were present in all 12 small-cell carcinomas of the urinary bladder, including an at least fivefold lower number of T cells in the CT as compared to the IM. This was in contrast to urothelial carcinomas, where the findings were more variable but dominated by characteristics of the “inflamed” cancer phenotype. Comparable absolute numbers on T cell density in “cells per mm^2^” are not available in the literature, but the inflamed phenotype had been attributed to urothelial bladder cancer before [15]. So far, immune exclusion has been mostly reported from colorectal and pancreatic adenocarcinomas [15]. It was also found in a large fraction of small-cell lung cancers (SCLC) [15–17]. That small-cell carcinoma of the urinary bladder is also characterized by immune exclusion which provides another argument for a considerable similarity between small-cell carcinomas of the lung and the bladder which had been earlier suggested by comparative studies analyzing genomic alterations [18, 19].

Tumors containing distinct areas of both the “inflamed” and the “immune-excluded” phenotype may serve as models to study the molecular mechanisms underlying immune exclusion. About 40% of small-cell carcinomas of the bladder show mixed tumor components of small-cell and urothelial cancers [1]. The close spatial relationship with unequivocal transition of both histological subtypes indicates that small-cell cancers typically develop from urothelial carcinomas. Studies using comparative genomic hybridization, next-generation sequencing, and immunohistochemistry have demonstrated that urothelial carcinoma progresses to small-cell cancer through accumulation of additional genomic alterations. These include both gross chromosomal changes such as deletions (1q31-qter, 2q14-qter, 3q, and 10q) and gains (1q21–q23, 10p) [18] as well as somatic mutations of the RB1 tumor suppressor and TERT promotor [19–21]. Our cohort contained two cases with presumable urothelial to small-cell cancer progression, and both cancers showed remarkably similar features. Both had high PD-L1 expression and high numbers of CD8^+^ lymphocytes at IM and in CT of the urothelial cancer component and a complete loss of PD-L1 expression and an almost complete loss of CT CD8^+^ lymphocytes in the small-cell cancer component. Those both cancers completely lost their strong and abundant PD-L1 expression which demonstrates how variable the PD-L1 status can be in tumors and that molecular progression can fundamentally change the role of PD-L1 in a given cancer.

These findings raise the hypothesis that the molecular progression to small-cell bladder cancer may quite regularly involve the acquisition of a new and strong ability to defend the tumor against lymphocytic infiltration. It could be speculated that already the compact growth pattern of small-cell cancers hampers immune cell infiltration, but small-cell bladder carcinomas may also develop established molecular immune escape mechanisms including physical or chemical barriers, such as extracellular matrix remodeling or TGF-beta-1 upregulation [11, 22–24]. Whatever the mechanism may be, the acquired immune-excluded phenotype [11, 12] is unlikely to be due to a loss of mutation-associated neoantigens [25, 26] because small-cell bladder carcinomas have a higher mutational burden than urothelial carcinomas and specific mutations can hardly be lost in cancers during progression [19]. It is possible that the immune escape occurring in small-cell carcinomas is so efficient that cancer cells do not further require PD-L1 expression. Interestingly, lack of PD-L1 expression has earlier been linked to “noninflamed” tumor phenotype and been reported to be common in small-cell cancers of the lung [12, 17, 27]. All these findings indicate that small-cell carcinomas of the urinary bladder may represent model tumors for immune oncology. The frequent cooccurrence of a morphologically defined inflamed cancer and its immune-excluded postprogression counterpart may be excellently suited to study the molecular mechanisms underlying downregulation of PD-L1 and the development of “immune exclusion.”

The immune-excluded and immune-desert phenotypes have initially been described as noninflamed [11, 12] microenvironments that might respond less favorably to immune checkpoint therapy than the inflamed phenotype [12]. However, there is accumulating evidence that the degree of lymphocytic infiltration at the IM might be more predictive for outcome than that in the CT. For example, higher lymphocytic infiltration at the IM than in the CT has been described particularly in tumor entities that often respond to anticheckpoint therapy [4, 28–32], such as non-small-cell lung cancer, squamous cell carcinoma of the head and neck, or renal cell carcinoma [33, 34]. Moreover, the amount of CD8^+^ cytotoxic T lymphocytes within the IM was the best predictive parameter for clinical response to checkpoint therapy in a study analyzing metastatic melanoma [9]. Our study demonstrates that most urothelial carcinomas have more CD8^+^ cytotoxic T cells at the IM than in the CT and that this particularly applies to small-cell bladder cancers. The typically high number of CD8^+^ at the IM of small-cell bladder cancers may explain a recent report of a patient with small-cell carcinoma of the bladder responding well to pembrolizumab [5].

In conclusion, the result of our study highlights immune exclusion as a key feature of small-cell bladder cancer. Progression of small-cell bladder cancer from urothelial cancer represents a model case of a near complete change in the immune environment going along with morphological—and most likely also—genomic dedifferentiation of bladder cancer. Small-cell bladder cancer may benefit from treatment strategies that are specifically designed to overcome immune exclusion.

## Figures and Tables

**Table 1:** Total T cell, cytotoxic T cell, and PD-1-positive T cell densities at the invasive margin (IM) and the center of the tumor (CT) in two patients with adjacent areas of small-cell and urothelial cell bladder cancer.

Patient	Small-cell part			Urothelial part		
	CD3^+^ (cells/mm^2^)	CD8^+^ (cells/mm^2^)	PD-1^+^ (cells/mm^2^)	CD3^+^ (cells/mm^2^)	CD8^+^ (cells/mm^2^)	PD-1^+^ (cells/mm^2^)
No. 8						
IM	1328	254	676	1355	628	764
CT	109	13	7	1063	823	545
No. 4						
IM	1526	394	873	1097	234	824
CT	50	23	38	466	78	385

## Data Availability

The data used to support the findings of this study are available from the corresponding author upon request.

## Supplementary Materials

Supplementary Materials Figure S1: CD3-, CD8-, and PD-1-positive cell densities of 12 patients with small-cell carcinoma of the bladder at the invasive margin (IM) and the center of the tumor (CT) (P1-P12).
