# Supplementary material for: Immune Exclusion Is Frequent in Small-Cell Carcinoma of the Bladder
Source: Dis Markers. 2019 May 2;2019:2532518. doi: 10.1155/2019/2532518 (PMC6525886; doi:10.1155/2019/2532518)
Supplement: Supplementary Materials — Figure S1: CD3-, CD8-, and PD-1-positive cell densities of 12 patients with small-cell carcinoma of the bladder at the invasive margin (IM) and the center of the tumor (CT) (P1-P12). [file 2532518.f1.pdf]

Supplementary information

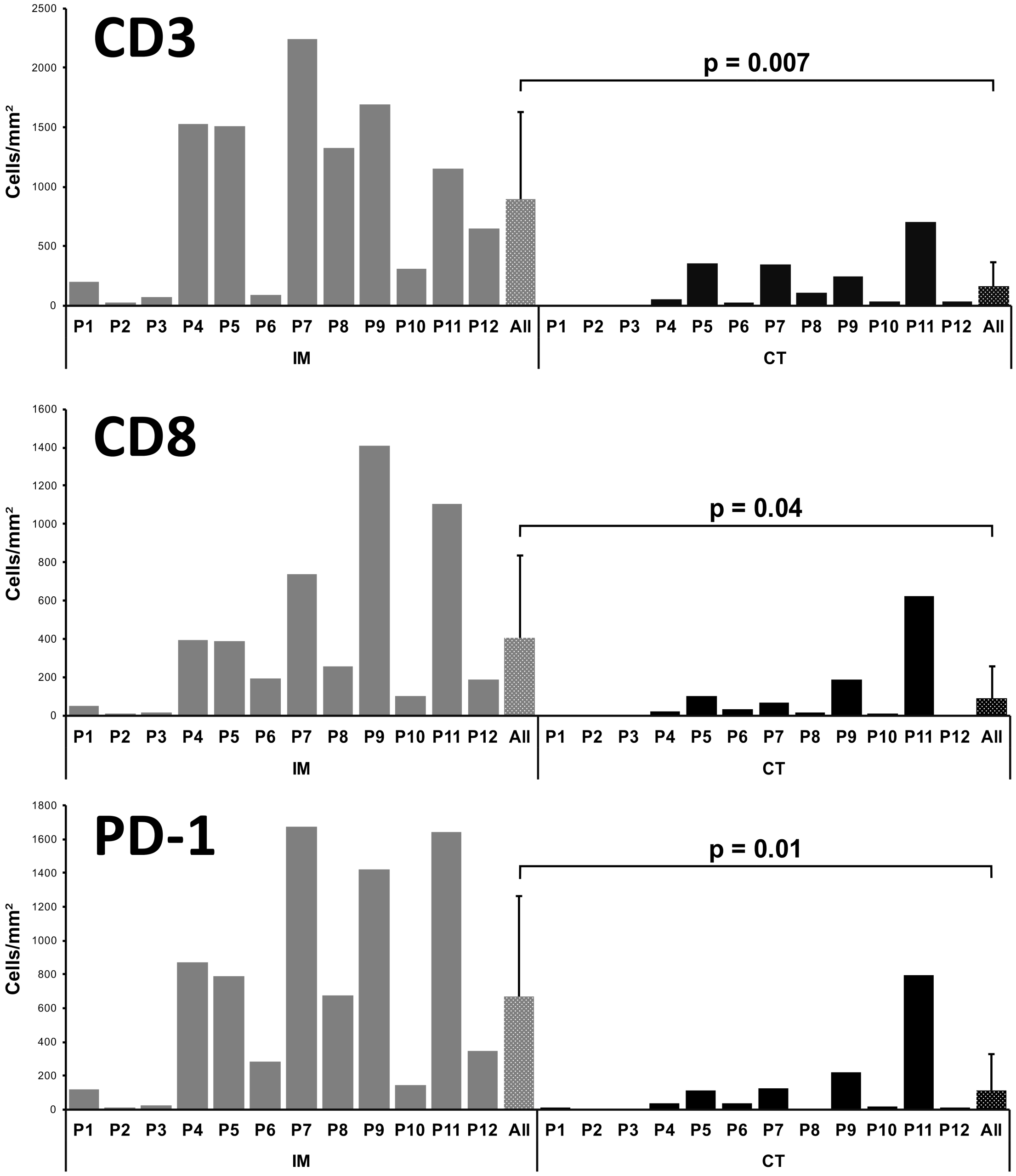

Figure S1. CD3, CD8 and PD-1 positive cell densities of 12 patients with small cell carcinoma of the bladder at the invasive margin (IM) and the center of the tumor (CT) (P1-P12).
